# Evolution of Smooth Tubercle Bacilli PE and PE_PGRS Genes: Evidence for a Prominent Role of Recombination and Imprint of Positive Selection

**DOI:** 10.1371/journal.pone.0064718

**Published:** 2013-05-21

**Authors:** Amine Namouchi, Anis Karboul, Michel Fabre, Maria Cristina Gutierrez, Helmi Mardassi

**Affiliations:** 1 Unit of Typing and Genetics of Mycobacteria, Laboratory of Molecular Microbiology, Vaccinology, and Biotechnology Development, Institut Pasteur de Tunis, Tunis, Tunisia; 2 Laboratoire de Biologie Clinique, HIA Percy, Clamart, France; 3 Départment Infection et Epidémiologie, Institut Pasteur, Paris, France; Fordham University, United States of America

## Abstract

**Background:**

PE and PE_PGRS are two mycobateria-restricted multigene families encoding membrane associated and secreted proteins that have expanded mainly in the pathogenic species, notably the *Mycobacterium tuberculosis* complex (MTBC). Several lines of evidence attribute to PE and PE_PGRS genes critical roles in mycobacterial pathogenicity. To get more insight into the nature of these genes, we sought to address their evolutionary trajectories in the group of smooth tubercle bacilli (STB), the putative ancestor of the clonal MTBC.

**Methodology/Principal Findings:**

By focussing on six polymorphic STB PE/PE_PGRS genes, we demonstrate significant incongruence among single gene genealogies and detect strong signals of recombination using various approaches. Coalescent-based estimation of population recombination and mutation rates (ρ and θ, respectively) indicates that the two mechanisms are of roughly equal importance in generating diversity (ρ/θ = 1.457), a finding in a marked contrast to house keeping genes (HKG) whose evolution is chiefly brought about by mutation (ρ/θ = 0.012). In comparison to HKG, we found 15 times higher mean rate of nonsynonymous substitutions, with strong evidence of positive selection acting on PE_PGRS62 (dN/dS = 1.42), a gene that has previously been shown to be essential for mycobacterial survival in macrophages and granulomas. Imprint of positive selection operating on specific amino acid residues or along branches of PE_PGRS62 phylogenetic tree was further demonstrated using maximum likelihood- and covarion-based approaches, respectively. Strikingly, PE_PGR62 proved highly conserved in present-day MTBC strains.

**Conclusions/Significance:**

Overall the data indicate that, in STB, PE/PE_PGRS genes have undergone a strong diversification process that is speeded up by recombination, with evidence of positive selection. The finding that positive selection involved an essential PE_PGRS gene whose sequence appears to be driven to fixation in present-day MTBC strains lends further support to the critical role of PE/PE_PGRS genes in the evolution of mycobacterial pathogenicity.

## Introduction

Tuberculosis (TB) still remains a huge threat for human and animal health worldwide [1]. TB is caused by members of the *Mycobacterium tuberculosis* complex (MTBC) which classically comprises *Mycobacterium tuberculosis*, *Mycobacterium bovis*, *Mycobacterium microti*, *Mycobacterium africanum*, *Mycobacterium pinnipedii* and *Mycobacterium caprae* species [2], [3]. Additional mycobacterial species, referred to as smooth tubercle bacilli (STB), including the species “*Mycobacterium canettii*” were also shown to cause TB [4], [5].

Although the MTBC members have shown a large spectrum of phenotypic characteristics and mammalian hosts, they tend to represent a genetically homogeneous group thought to have emerged recently [6], [7], [8]. Indeed, genetic analyses and comparative genomics provided evidence that the MTBC group may have descended from a single successful clone following an evolutionary bottleneck that occurred 20 000 to 40 000 years ago [2], [7], [9], [10]. Surprisingly, the STB group showed an unprecedented high level of genetic diversity. The distribution of the nucleotide polymorphism in hsp65 gene sequence raised the possibility that MTBC could have emerged from *M. canettii* [11]. Gutierrez et al. [12] expanded upon this finding by carrying out a multilocus sequence analysis leading to the suggestion that STB correspond to pre-bottleneck lineages, and may thus represent members of a much broader progenitor species, named *M. protototuberculosis*, from which the MTBC clonal group could have evolved. The question whether MTBC indeed arose from members of the STB group [13], [14], [15], [16] was thoroughly addressed by sequencing the whole genomes of five representative strains of STB [17]. The findings strongly confirm that *M. tuberculosis* emerged from an ancestral STB-like pool of mycobacteria. Hence, exploring the biology of STB would be of interest to trace back early events in the evolution of MTBC.

A hallmark of the genomes of pathogenic mycobacteria is the abundance of two large multigene families, PE and PPE, named after their N-terminal Pro-Glu (PE) or Pro-Pro-Glu (PPE) motifs [7]. PE/PPE genes encode membrane, surface exposed, and/or secreted proteins, involved in many facets of the interaction with the host [18], [19], [20]. Phylogenetic analyses showed that these gene families are restricted to mycobacteria and accompanied their evolution [21]. Of particular interest, the highly repetitive subfamilies PE_PGRS and PPE_MPTR expanded only in the genome of the MTBC members and close relatives. Consistently, we have also identified a polymorphism in a duplicated PE_PGRS gene pair, whose distribution through members of the MTBC, and several other mycobacterial species, provided an evolutionary history that conforms to the established scenario and confirmed the ancient origin of the smooth tubercle bacillus, *M. canettii* [22].

A myriad of roles have been attributed to PE/PPE genes, all converging towards critical functions in the bacillus's interaction with the host. It has been speculated that the relative polymorphic nature of their coding sequence may promote immune evasion, through antigenic variation [7], [8], [23], [24], [25], [26], [27], [28], [29], [30], [31], inasmuch as PE/PPE genes encode for secreted or cell surface exposed proteins that elicit protective immune responses [32], [33], [34], [35], [36], [37], [38], [39]. However, such assumption awaits experimental validation. By contrast, there is some experimental evidence that points to a prominent role of PE/PPE proteins, notably of the PE_PGRS and PPE_MPTR subfamilies, in modulating the macrophage function, allowing the bacillus to establish a successful infection. The first evidence came from Ramakrishnan et al. [40] who demonstrated the essential role of PE_PGRS62 in survival of *M. Marinum* inside macrophages and persistence in the granuloma. Soon thereafter, the role of PE_PGRS33 in promoting macrophage uptake was established [41]. Additional studies have further confirmed that several PE/PE_PGRS/PPE members are required for survival in macrophages and in mice [42], [43], [44], [45], [46]. The finding that a number of PE_PGRS and PPE genes were involved in the modulation of phagocytosis, is consistent with their critical role in host defence subversion [47], [48], [49]. Furthermore, PE/PPE proteins, and PE_PGRS in particular, were found to be implicated in the modulation of both innate and adaptive immune responses, as well as in diverse aspects of the infection process [18], [19], [20], [50], [51], [52], [53].

Since PE/PPE genes expanded in pathogenic mycobacteria, especially MTBC and close relatives, one can rightfully argue that they could have contributed to their pathogenicity. Here we focussed on the highly repetitive PE/PE_PGRS subfamily, since it has been assigned multiple roles in virulence and modulation of the host immune response. We hypothesized that exploring the evolutionary trajectories of PE/PE_PGRS genes in the ancestral STB group, the putative progenitor of MTBC, may provide new insights into their importance in the evolution of mycobacterial pathogenicity.

The results presented here showed that both recombination and mutation impacted the evolution of PE/PE_PGRS in STB. In comparison to house keeping genes, recombination accelerated the diversification process of PE/PE_PGRS genes allowing selection to operate. We also present an obvious example of positively selected PE_PGRS gene, whose sequence is likely to be fixed in present-day MTBC strains, consistent with its critical pathogenic role.

## Materials and Methods

### Ethics statement

This study involved only DNA from Mycobacteria that have previously been described and published. No sputum or any other samples were collected from patients for the specific needs of this study.

### Mycobacterial isolates

MTBC strains used in this study included H37Rv (TubercuList; http://genolistpasteurfr/TubercuList/), H37Ra [54], CDC1551 [8], a Haarlem3 Tunisian MDR outbreak strain [55], *M. bovis* strain AF2122/97 (BoviList; http://genolistpasteurfr/BoviList/), BCG Pasteur 1173P2 (BCGList; http://genolist.pasteur.fr/BCGList/), 1 *M. africanum* (type strain; CIPTB 140030001 of the Institut Pasteur collection), 1 *M. microti* (type strain, CIPTB 140050001 of the Institut Pasteur collection) and 1 *M. pinnipeddii* (CIPTB 140090001 of the Institut Pasteur collection). The collection of STB contained 28 strains covering the nine genotypes, A to I (1 A, 20 C/D, 1 B, 1 E, 1G, 2 H, 1 F and 1 I), described earlier by Gutierrez et al. [12]. Details regarding the origin and year of isolation of the mycobacterial isolates are listed in Table S1.

### PCR amplification and DNA sequencing

Three PE (PE3, PE4, and PE35) and 6 PE_PGRS (PE_PGRS12, PE_PGRS26, PE_PGRS29, PE_PGRS35, PE_PGRS51, and PE_PGRS62) genes scattered throughout the *M. tuberculosis* H37Rv genome were selected. The sequence of the primers used for PCR amplification and sequencing of each gene fragment is provided in Table S2. The specificity of each primer was checked using the updated TubercuList database (http://tuberculist.epfl.ch/) [56]. Although the genome sequence of some reference strains is publicly available, we PCR amplified and sequenced the PE/PE_PGRS selected members from the DNA of all these strains, since the accuracy of genome sequences in these highly GC-rich and repetitive regions is questioned.

The amplification reaction mixture contained 20 ng of template genomic DNA, 10 µl of 10x buffer (Qiagen), 10 µl DMSO, 2 µl of 10 mM nucleotide mix (Amersham Biosciences), 2 µl of each primer (20 µM stock), 0, 25 µl (1.25 U) of HotStar *Taq* DNA polymerase (Qiagen) and sterile nuclease-free water (Amersham Biosciences) to 50 µl total reaction volume. Cycling was carried out in a 2720 thermocycler (Applied Biosystems) with an initial denaturation step of 10 min at 96°C followed by 35 cycles consisting of 1 min at 95°C, 1 min at 60°C and 2 min at 72°C. The amplification ended with a final elongation step of 7 min at 72°C.

Amplicons were subjected to sequencing after treatment with Exonuclease I (Amersham Biosciences) and Shrimp Alkaline Phosphatase (Amersham Biosciences). The reaction consisted of 1.5 µl of BigDye terminator cycle sequencing reagents, 4 µl of BigDye terminator cycle sequencing buffer, 1 µl of 20 µM concentrations of primers, as well as sufficient UltraPURE Distilled DNase, RNase-Free Water (Gibco/Invitrogen) to make a 20-µl reaction. Cycle sequencing was performed using a 2720 thermocycler (Applied Biosystems) programmed for 25 cycles at 96°C for 10 s, 50°C for 5 s, and 60°C for 4 min. The template DNA was ethanol-precipitated, washed, and subjected to automated sequencing on an ABI Prism 3130 genetic analyzer (Applied Biosystems) according to the manufacturer's protocol.

Both strands of each amplicon were sequenced from two independent PCR amplification reactions.

### Genetic polymorphism and diversity

The DnaSP software package, version. 4.10 [57] was used to carry out several population genetic analyses. For each locus, we determined the number of haplotypes (h), number of polymorphic sites (S), nucleotide diversity (π), and the per-site population mutation rate, θ (2N_e_μ). To test for adaptive selection, we determined the nucleotide substitution changes and the ratio of nonsynonymous (dN) to synonymous (dS) substitutions per site (dN/dS), using the analysis developed by Nei-Gojobori [58] after Jukes-Cantor correction for multiple substitutions.

### Phylogenetic analysis

Phylogenetic relationships were reconstructed by taking into account all polymorphic sites. To assess the possible confounding effect of positive selection [59], we compared the obtained trees with those built with synonymous changes only. Analyses were either performed on a single gene basis or on the concatenated sequence of the six most variable PE/PE_PGRS genes (PE3, PE4, PE_PGRS26, PE_PGRS35, PE_PGRS51, and PE_PGRS62). Alignments of gene sequences were performed using the ClustalW program [60].

Maximum likelihood (ML) methods were used to infer phylogenetic relationships for each of the six most variable PE/PE_PGRS genes, as well as for their concatenated sequence. ML analyses were performed using RaxML version 7.2.8 [61]. Bootstrap confidence levels were based on 1000 resampling. The trees were visualized using FigTree (http://tree.bio.ed.ac.uk/software/figtree/).

Prior to ML analyses, a DNA substitution model for each data set was selected. To determine the best substitution model, we compared the results of the TOPALi v2 package [62] with those of the Hyphy-package that is available through the web site www.datamonkey.org [63]. Two different scores, Akaike information criterion (AIC) and Bayesian information criterion (BIC), have been calculated in order to determine, for each data set, the best model. The two analyses yielded the same results. The best substitution models obtained with TOPAli are provided in the File S1. For the majority of the data sets the model F81 [64] was determined to be optimal.

To test for the topological congruence between trees, we computed the *Icong* index, which is based on maximum agreement subtrees (MAST) [65]. This method determines the minimum number of leaves that have to be removed in each phylogeny to render the trees identical. Computation of *Icong* and of the associated *P*-value was performed on line at http://max2.ese.u-psud.fr/bases/upresa/pages/devienne.

### Tests of recombination

Because signals of positive selection and recombination may be confounded [59], only synonymous substitutions were considered to test for recombination. Alignments were screened for evidence of recombination in both PE/PE_PGRS and HKG by using a combination of different methods since the detection abilities of different tests can vary markedly [66]. We assume that the use of multiple tests, which are based on different approaches, allows a more robust prediction of the occurrence of recombination.

First, we performed a split decomposition analysis [67] to generate phylogenetic networks. The networks were generated in Splitstree 4.6 [68], and evidence of recombination, indicated by the presence of cycles in the networks, was assessed by the pairwise homoplasy index (PHI). Significance of the PHI statistic is assessed with the normal approximation of a permutation test where, under the null hypothesis of no recombination, sites along the alignment are randomly permuted to obtain the null distribution of PHI. *P* values<0.05 indicate significant presence of recombination [69].

Further we used several other recombination detection algorithms:

- **Hudson and Kaplan's** ***R*** **_min_** [70]: The Hudson and Kaplan's lower bound on the minimal number of recombination events in an infinite site model was computed using DnaSP v4.0 program [57].
- **Maximum chi-square** [71]: A nonparametric method that detects regions of the aligned sequences delimited by putative recombination breakpoints. Maximum chi-square is a component of the software package RDP 2.0 [72].
- **The Web-based service GARD** (genetic algorithm for recombination detection) [73]: a model-based approach that searches for putative breakpoints delimiting sequence regions having distinct phylogenies. Briefly, GARD compares a nonrecombinant model in which the sequence data are fitted to a single phylogeny to models in which breakpoints partition the sequence data into two or more regions having varying phylogenies. Site-by-site substitution rate was assumed to be constant between sites. The identified breakpoints were further confirmed using the akaike information criterion (AIC) score and Kishino-Hasegawa topological incongruence test.
- **Recco** [74]: A sensitive method for detecting recombination events, based on a cost minimization approach. The probability of detecting recombination was adjusted such as to classify a dataset as recombinant if the *P*-value does not exceed 0.12 (MaxSavings feature of Recco). The mutation cost matrix is set to Hamming, which means that for any a and b characters m(a,a) = 0 and m(a,b) = 1 for any a! = b.

**Estimation of the per-locus population recombination rates**

The program pairwise from the LDhat package [75] was used to obtain an approximate-likelihood estimate of the population recombination rate (2N_e_r), ρ, by combining the coalescent likelihoods of all pairwise comparisons of segregating sites. The per-site population mutation rate (2Neμ) was estimated using the Watterson's estimator of Theta implemented in the LDhat package. This program is accessible through http://www.stats.ox.ac.uk/~mcvean. PE_PGRS62 was omitted from these analyses due to evidence that it might be under positive selection.

### Tests of selection

Evidence of positive selection in a protein's amino acid sequence is generally indicated by an excess of nonsynonymous substitutions relative to synonymous substitutions, the dN/dS ratio (or *ω*). Evidence of positive selection for amino acid replacements is suggested when *ω*>1, purifying selection is inferred when *ω*<1, whereas neutral evolution is assumed when *ω* = 1. First we measured the *ω* over the entire length of a gene, then we performed a codon-by-codon analysis using codeml as implemented in the software package PAML (Phylogenetic Analysis by Maximum Likelihood) v. 4.4e [76]. For this purpose we used “site models” where codon sites are allowed to fall into categories depending on their *ω* values. First, we compared a “nearly neutral model”, M1a, to a “positive selection” model, M2a. The model M1a allows 2 categories of codon sites in *p*_0_, and *p*_1_ proportions, with *ω*_0_<1 and, *ω*_1_ = 1, whereas M2a adds an additional category of codons (*p*_2_), with *ω*_2_ that is free to vary above 1. In addition to M1a and M2a, we compared several additional site models, M7, M8, and M8a. M7 specifies a neutral model similar to M1a, but the sites affected by negative selection approximate a beta distribution with parameters (*p* and *q*) estimated from the data. M7 is compared to M8 (selection) for which the category of sites with a dN/dS>1 is added. We also compared the model M8 to M8a. In the latter model the extra *ω* is fixed at 1. Previous studies have shown that the M8–M8a comparison is more robust than the M7–M8 comparison and produces less false positives [77], [78].

The comparison between models was assessed using Likelihood-Ratio Tests (LRTs). A significantly higher likelihood of the alternative model than that of the null model indicating positive selection in the data set examined. For models comparisons, we used degree of freedom, df = 2. For each analysis, correction for multiple testing (Bonferroni correction) was applied. Only in cases where LRT was significant, we used the Bayes empirical Bayes (BEB) procedure to calculate the posterior probabilities (PPs) to identify sites under positive selection [79].

Signals of positive selection were also searched along all branches of the constructed phylogenetic trees. For this purpose we used a parsimony approach for ancestral sequence reconstruction [80], coupled with a covarion-based approach for the calculation of dN/dS ratios [81].

### Nucleotide sequence accession numbers

STB PE/PE_PGRS sequences obtained in this study were deposited in the EMBL database under accession numbers HE855688 to HE855735. For comparative analyses, nucleotide sequences of the house keeping genes (HKG), *gyr*A, *gyr*B, *hsp*65, *kat*G, and *rpo*B, generated in the study of Gutierrez et al. [12], were used.

## Results

### Genetic diversity of PE/PE_PGRS genes

The 3 PE (PE3, PE4, and PE35) and 6 PE_PGRS (PE_PGRS12, PE_PGRS26, PE_PGRS29, PE_PGRS35, PE_PGRS51, and PE_PGRS62) genes were selected for sequencing in STB, since they could be reliably amplified by PCR in both MTBC and STB.

Estimates of parameters for DNA divergence are summarized in Table 1. Sequence diversity was about 5-fold greater in the STB group with a mean nucleotide diversity (π x 100) of 0.398 compared to 0.08 in the MTBC group. Likewise, the average per-site population mutation rate (θ×100) was nearly 4 times higher in STB than in MTBC (0.406 vs 0.103). In the latter group, 3 out of the 9 selected PE/PE_PGRS genes proved conserved.

**Table 1:** Nucleotide diversity and summary statistics.

Gene	Gene Length (pb)	Sequenced region	MTBC/STB			
			h^a^	S^b^	π^c^	θ^d^
PE3 (Rv0159c)	1407	+58 to+1371	3/5	2/12	0.00042/0.00342	0.00054/0.00352
PE4 (Rv0160c)	1509	+31 to+1461	4/6	3/17	0.00085/0.00482	0.00074/0.00485
PE_PGRS26 (Rv1441c)	1476	+31 to+1443	6/5	19/21	0.00332/0.00632	0.00536/0.00573
PE_PGRS35 (Rv1983)	1677	+31 to+1650	3/5	2/10	0.00027/0.00212	0.00045/0.00212
PE_PGRS51 (Rv3367)	1767	+31 to+1737	5/8	4/40	0.00076/0.00858	0.00083/0.00904
PE_PGRS62 (Rv3812)	1515	+31 to+1485	1/6	0/14	−/0.00353	−/0.00371
PE35 (Rv3872)	300	+1 to+300	2/3	1/3	0.00159/0.00464	0.00136/0.00386
PE_PGRS12 (Rv0832)	414	+31 to+369	1/3	0/3	−/0.00222	−/0.00342
PE_PGRS29 (Rv1468c)	1113	+31 to+1074	1/2	0/1	−/0.00024	−/0.00037
Mean value	-	-	-	-	0.00080/0.00398	0.00103/0.00406

a number of haplotypes.

b number of polymorphic sites.

c Nucleotide diversity.

d population mutation rate. Per site Waterson's θ, 2Neμ.

No sequence variation could be observed among STB strains of the same genotype. Indeed, the nucleotide sequence was identical among the 20 strains of genotype C/D. Moreover, the couple of strains within genotype H showed no variation. The most variable loci, each showing more than 10 polymorphic sites among STB genotypes, were PE3, PE4, PE_PGRS26, PE_PGRS35, PE_PGRS51, and PE_PGRS62. PE_PGRS51 displayed the highest level of sequence variability (40 polymorphic sites). Of note, although the sequence of PE_PGRS62 varied significantly in the STB group (14 polymorphic sites), it showed no polymorphism among the MTBC strains used in this study. Therefore we extended our analysis to 25 additional clinical *M. tuberculosis* isolates belonging to different spoligotype families and originating from various geographic areas (Table S3). Intriguingly, the nucleotide sequence of PE_PGRS62 proved highly conserved. Only a single sSNP could be detected in a Tunisian clinical isolate of the Haarlem genotype (data not shown).

### Phylogenetic relationships between STB genotypes based on PE/PE_PGRS polymorphism

**Figure 1:**
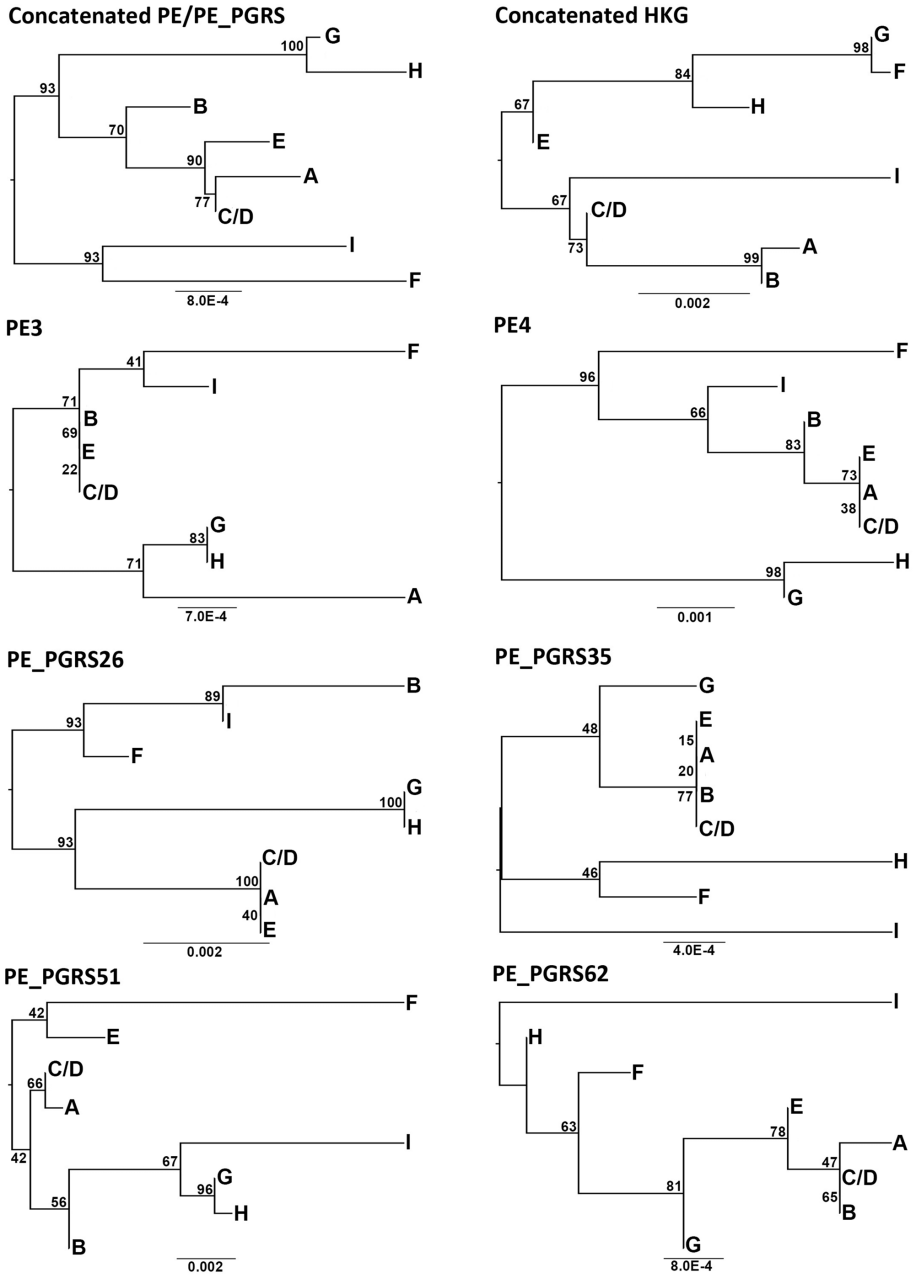
ML Phylogenetic trees showing the relationships of STB genotypes based on PE_PGRS and HKG polymorphism. HKG sequences were imported from the study of Gutierrez et al. [12]. Numbers on branches of the ML tree are bootstrap support values.

To establish the phylogenetic relationship between STB genotypes, we constructed a maximum likelihood (ML) tree based on the concatenated sequence of the most variable six PE/PE_PGRS genes (Fig. 1). The 8 genotypes could be subdivided into two main branching groups. Genotypes corresponding to *M. canettii* (A and C/D) grouped together and were phylogenetically closely related to genotypes E and B. Genotypes G and H, although deriving from the same phylogenetic branch appeared distantly related to the above group (*M. canettii* and genotypes E and B). The remaining two genotypes, I and F, proved distantly related to the other genotypes as they formed a distinct genetic branch. The ML tree based on HKG polymorphism (Fig. 1B) was incongruent with that obtained with PE/PE_PGRS genes (*Icong* = 1.226; not significant).

### Evidence of intragenic and intergenic recombination among PE/PE_PGRS genes

We tested for evidence of recombinant genotypes by evaluating whether the genealogical relationships described by each locus were congruent. For this purpose, we performed a ML phylogenetic analysis on each of the six variable PE/PE_PGRS genes (Fig. 1). A clear difference in both tree topologies and branch lengths could be observed between the six phylogenies. For instance, the genotypes corresponding to *M. canettii* (A and C/D) appeared distantly related in PE3- and PE_PGRS62-ML based trees. On the other hand, genotype B, which is closely related to *M. canettii*, tended in some phylogenies to be more related to the divergent genotype I (PE_PGRS26- and PE_PGRS51-ML based trees). Computation of *Icong* and its associated *P*-value further confirmed the marked conflict among individual gene phylogenies (Table 2). Indeed incongruence was observed in 80% of 15 pairwise comparisons among the six variable PE/PE_PGRS loci. Likewise, conflicts between single gene phylogenies and the concatenated sequence-based ML tree were noted in 83.33% of pairwise comparisons.

**Table 2:** Conflict among tree topologies for different loci as assessed by the maximum agreement subtrees (MAST) method.

	PE3	PE4	PE_PGRS26	PE_PGRS35	PE_PGRS51	PE_PGRS62
PE4	0.98 (3.709)					
PE_PGRS26	1.226 (0.190)	**1.472 (0.009)**				
PE_PGRS35	0.981 (3.709)	0.736 (2.08)	0.981 (3.709)			
PE_PGRS51	0.981 (3.709)	1.226 (0.19)	1.226 (0.190)	1.226 (0.190)		
PE_PGRS62	0.736 (2.08)	**1.717 (5×10^−4^)**	0.981 (3.709)	**1.472 (0.009)**	0.981 (3.709)	
Concatenate	1.226 (0.190)	0.981 (3.709)	**1.717 (5×10^−4^)**	0.981 (3.709)	0.981 (3.709)	0.981 (3.709)

Significant congruence is indicated in bold. Note that incongruence occurs in 80% (3/15) of pairwise comparisons among the six PE/PE_PGRS genes. Numbers indicate the *Icong* value followed by (*P* value).

**Figure 2:**
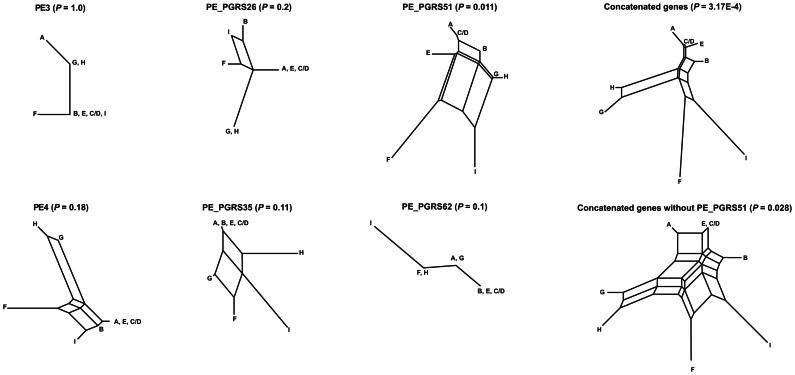
Split decomposition analysis of the six variable STB PE/PE_PGRS genes as well as their concatenated sequence. The *p*-value of the PHI test is indicated for each splitgraph. The analysis was performed by taking into account sSNPs only.

We further confirmed the above observations by constructing split decomposition networks of the six variable PE/PE_PGRS genes considering only sSNPs. Although the presence of cycles in the constructed networks could be observed for the majority of single gene phylogenies, statistically significant evidence for recombination could only be detected in PE_PGRS51 (PHI *P* value = 0.011) (Fig. 2). This finding is consistent with the ML phylogenetic analysis, as PE_PGRS51 showed no congruence with any other single gene phylogeny in the pairwise comparisons. Similar results were obtained when both nsSNPs and sSNPs were taken into account (data not shown).

We also investigated split decomposition networks based upon a concatenated alignment of the six variable PE/PE_PGRS genes. Evidence for recombination was supported by highly significant PHI test values (*P* = 3.17E−4; Fig. 2). The PHI test was still significant (*P* = 0.028) after eliminating the PE_PGRS51 sequence, the only gene that showed consistent evidence of recombination.

Overall, the above phylogenetic analyses provided strong signals of both intragenic (within PE_PGRS51) and intergenic recombination in STB PE/PE_PGRS genes. To further support these findings, we performed additional recombination detection tests. As recommended in previous studies [66], [82], we used a combination of various methods (Hudson and Kaplan's *Rmin*, Maximum chi-square, Recco, and GARD) since the detection abilities of different tests can vary markedly for a given dataset. The results obtained with the various methods are compiled in Table S4.

**Figure 3:**
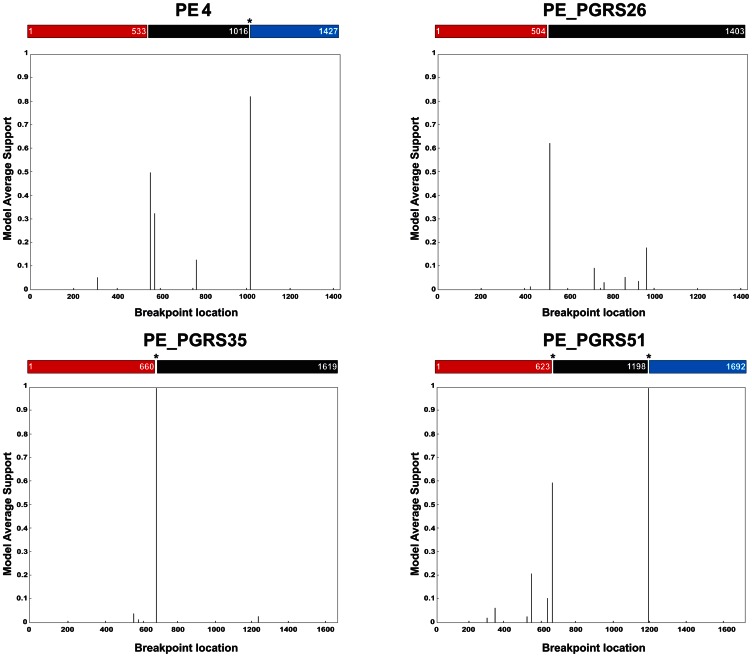
Detection of recombination breakpoints within PE/PE_PGRS sequences using GARD. The plots display potential recombination breakpoints within PE/PE_PGRS sequences. The probability of the breakpoints is evaluated by akaike information criterion (AIC) score and Kishino-Hasegawa topological incongruence test [73]. Model supported breakpoints are indicated with an asterisk.

**Figure 4:**
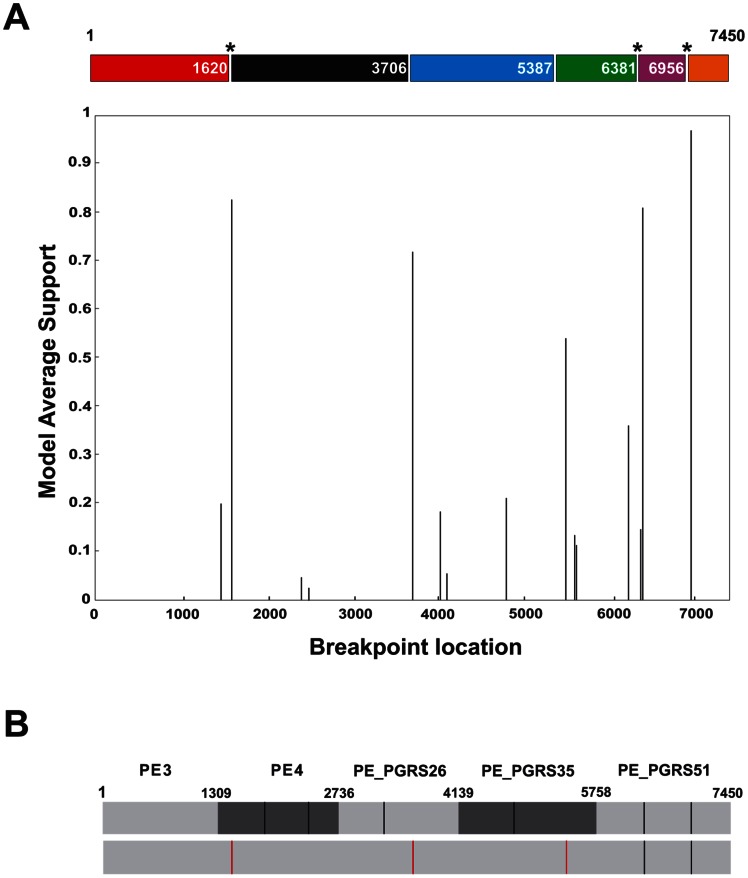
Detection of recombination breakpoints with GARD upon concatenation of PE/PE_PGRS sequences. (A) GARD plot showing potential recombination breakpoints within the concatenated PE/PE_PGRS sequence (PE3-PE4-PE_PGRS26-PE_PGRS35-PE_PGRS51). (B) Position of potential recombination breakpoints (black vertical bars) identified on a gene-by-gene analysis (top) and within the concatenated sequence (red vertical bars) (bottom). The probability of the breakpoints is evaluated by akaike information criterion (AIC) score and Kishino-Hasegawa topological incongruence test [73]. Model supported breakpoints are indicated with an asterisk.

Computation of the Hudson and Kaplan's *R*_min_ revealed six recombination events in the gene by gene analysis. When the concatenated sequence was assessed, four new recombination events were detected, thus pointing to the occurrence of intergenic recombination. Maximum chi-square test found no evidence of recombination within any of the PE/PE_PGRS genes, but did when the concatenated sequence was inspected, a finding consistent with intergenic recombination. Recco significantly identified recombination within PE_PGRS51, but failed to do so, neither in the other genes, nor in the concatenated sequence. Strikingly, GARD identified several breakpoints with a significant average model support (Fig. 3), some of which remarkably fit with the recombination events disclosed by the Hudson and Kaplan's method (Table S4). Three new breakpoints (nucleotide positions1620, 3706, and 5387) were identified by GARD in the concatenated sequence, providing an additional proof in favour of intergenic recombination (Fig. 4). The breakpoint at position 1620 was statistically significant and is very likely since it involved two neighbouring and homologous PE genes, PE3 and PE4.

When all the above methods were applied to STB HKG genes, significant signals of recombination could be demonstrated only for their concatenated sequence (data not shown).

### Estimation of the relative rates of recombination to mutation

Multilocus sequence typing proved suitable to measure the relative rate of recombination to mutation, a parameter believed to be of importance when studying gene diversification in bacteria [83]. We measured the relative contribution of recombination and point mutation to the diversification of PE/PE_PGRS genes within the population of STB using the coalescent-based method of McVean et al. [75], and compared it to that of HKG. The ratio of recombination to mutation as estimated by the ρ/θ ratio across the variable PE/PE_PGRS genes varied between 0.207 (PE3) to 2.946 (PE_PGRS35), yielding an average rate per locus of 1.48, which is far higher than the average rate of 0.0016 estimated for HKG (Table 3). This result suggests that in STB, recombination is roughly as important as mutation in generating diversity within a PE/PE_PGRS locus, a finding in a marked contrast to HKG whose evolution is chiefly brought about by mutation.

**Table 3:** Population recombination rate vs population mutation rate.

PE/PE_PGRS genes							House keeping genes					
	PE3	PE4	PE_PGRS26	PE_PGRS35	PE_PGRS51	Mean value	*gyr*A	*gyr*B	*hsp*65	*kat*G	*rpo*B	Mean value
ρ^a^ x 10^−3^	0.25	1.323	3.085	3.35	3.847	2.371	0.02	0.035	-	0.04	-	0.031
θ^b^ x10^−3^	1.205	1.685	1.6	1.137	2.508	1.627	3.262	2.905	-	1.288	-	2.485
ρ/θ^c^	0.207	0.785	1.928	2.946	1.534	1.457	0.00613	0.012	-	0.031	-	0.012

a ρ  = 2Ner.

b θ  = 2Neμ.

c ρ/θ: ratio of recombination to mutation rate.

### Evidence of positive selection

The first indication of adaptive selection is suggested by the fact that, in STB, PE/PE_PGRS genes accumulated 15 times more nonsynonymous substitutions (nsSNP) than did HKG (dN mean value of 0.003 vs 0.0002, respectively) (Table 4). By contrast, the mean value of the rate of synonymous substitution (dS) in HKG proved 2.6-fold higher compared to that of PE/PE_PGRS genes. The dN/dS ratio over all codon sites for each one of the six most variable PE/PE_PGRS genes was mostly<1 with a mean value of 0.438 (Table 4). Only for a single gene, PE_PGRS62, was the dN/dS greater than 1 (1.422), indicating that it is subject to positive selection.

**Table 4:** Estimation of synonymous (sSNP) and nonsynonymous (nsSNP) changes rates.

PE/PE_PGRS genes	nsSNPs	sSNPs	dN	dS	dN/dS
PE3	5	7	0.002 (0–0.0042)	0.007 (0–0.0202)	0.262
PE4	6	11	0.002 (0–0.0048)	0.012 (0–0.268)	0.205
PE_PGRS26	10	11	0.004 (0–0.0070)	0.011 (0–0.175)	0.381
PE_PGRS35	3	7	0.001 (0–0.0026)	0.006 (0–0.011)	0.110
PE_PGRS51	16	24	0.005 (0–0.0098)	0.019 (0.0021–0.0364)	0.252
PE_PGRS62	11	3	0.004 (0–0.0085)	0.003 (0–0.0078)	1.422
Mean value	-	-	0.003	0.009	0.438

Because the apparent purifying selection acting on the other PE/PE_PGRS genes may be masking positive selection of a few codons, we performed a codon by codon maximum likelihood test using the program codeml (PAML package). Only for PE4 and PE_PGRS62, was the likelihood ratio test close to significance for both models comparisons M1a vs M2a and M8a vs M8 (data not shown), and a few amino acid sites were identified under positive selection with Bayes empirical Bayes (BEB) analysis (Table 5). The leucine residue on position 115 of PE4 was identified by both models comparisons with a BEB posterior probability>95% (Table 5). In PE_PGRS62, the three amino acid sites (106Q, 253N, and 307Q) undergoing positive selection were identified only in the conservative M8–M8a comparison with BEB posterior probability values close to 95%. The three positively selected residues map to the highly repetitive PGRS part of PE_PGRS62.

**Table 5:** Likelihood scores and parameter estimates for STB PE/PE_PGRS genes assuming the F3x4 model of codon frequencies.

Model^a^	Log Likelihood	Parameter estimates^a^	Positively selected sites (BEB)^b^
**PE4**			
M1a: neutral	−1975.596593	*p*0 = 0.91590, *p*1 = 0.08410	
M2a: selection	−1969.253305	*p*0 = 0.99765, *p*1 = 0.00000,	115 L (0.958)
		*p*2 = 0.00235, ω2 = 10.68754	
			
M7: beta	−1975.658356	*p* = 0.005, *q* = 0.04827	
M8a: beta and ω = 1	−1975.596586	*p_0_* = 0.91591, *p_1_* = 0.08409,	Not allowed
		*p* = 0.00500, *q* = 1.05450	
M8: beta and ω	−1969.286308	*p*0 = 0.99768, *p* = 0.18993	115 L (0.962)
		*q* = 2.31635, ω = 11.53154	
			
**PE_PGRS62**			
M1a: neutral	−1966.440204	*p*0 = 0.50190, *p*1 = 0.49810	
M2a: selection	−1960.179961	*p*0 = 0.95359, *p*1 = 0.00000,	
		*p*2 = 0.04641	
		ω0 = 0.00000, ω1 = 1.00000,	
		ω2 = 28.99320	
			
M7: beta	−1967.313763	*p* = 0.43253, *q* = 0.00500	
M8a: beta and ω = 1	−1966.440201	*p_0_* = 0.50190, *p_1_* = 0.49810,	
		*p* = 0.00500, *q* = 1.36371	
M8: beta and ω	−1960.179961	*p*0 = 0.95359, *p* = 0.00500,	106 Q (0.943)
		*q* = 85.54963, ω = 28.99244	253 N (0.942)
			307 Q (0.944)

a *p* and *q* are parameters of the beta distribution. *p_0_*  =  proportion of sites falling into nearly neutral site class, *p*_1_ =  proportion of sites falling into neutral site class, *p*_2_  =  proportion of sites falling into positively selected site class.

b Sites (relative to H37Rv'corresponding gene numbering) falling into positively selected class with BEB estimates>0.90 are listed.

**Figure 5:**
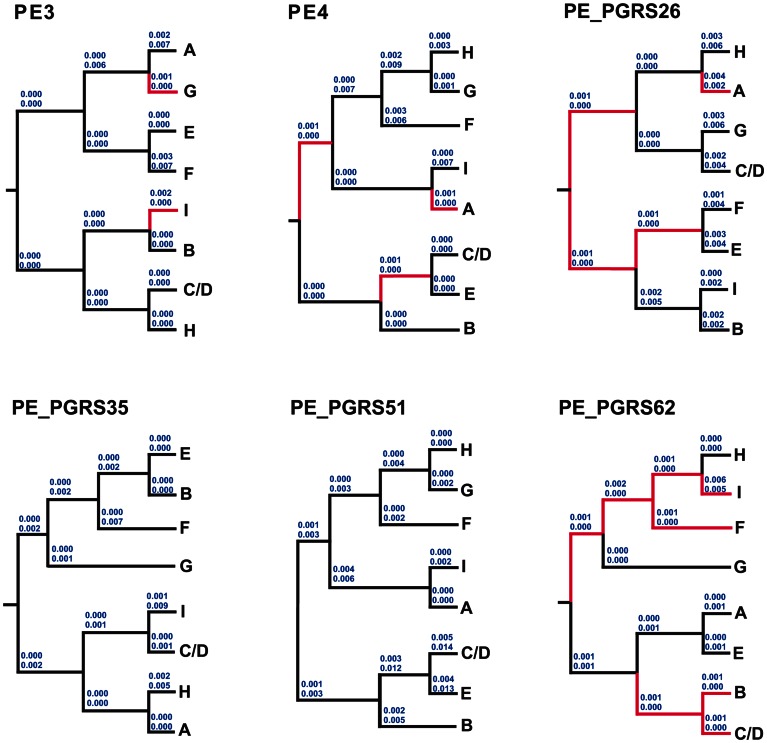
Estimation of dN/dS ratios along branches of STB PE/PE_PGRS individual trees using the covarion-based approach described by Siltberg and Liberles [80]. dN and dS rates (top and bottom numbers, respectively) are shown on branches. Branches where positive selection was detected are drawn in red.

Finally, we searched for signals of positive selection along specific branches of STB PE/PE_PGRS phylogenetic trees. For this purpose we reconstructed ancestral sequences and calculated dN/dS ratios along all branches using a covarion-based approach [81]. Imprint of positive selection acting along specific branches was evident for PE_PGRS62 (Fig. 5, red branches), confirming PAML hypothesis testing. PE_PGRS62 was undergone positive selection very early since the common ancestor, and in some cases (genotypes I and F), the selective pressure was maintained throughout its evolution. Positive selection acting on specific branches of PE3, PE4 and PE_PGRS26 was detected, albeit with very low dN values (0.001–0.002), most likely due to the fact that the covarion-based approach only samples sites that are potentially under selective pressure [80].

## Discussion

PE/PE_PGRS genes encode surface-exposed and/or secreted proteins involved in many facets of the interaction with the host [20]. Previous phylogenetic studies indicated that PE/PE_PGRS genes mainly expanded in the pathogenic MTBC group and accompanied its evolution [21]. In the present study we examined their molecular evolution in STB, the mycobacterial lineage that most likely represents the ancestor of the MTBC.

We show that the molecular mechanisms driving PE/PE_PGRS evolution in STB are significantly different from those operating on HKG, a finding reflected in the incongruence of their respective tree topographies. Indeed, while HKG evolved mainly by mutation, the pattern of polymorphism in PE/PE_PGRS genes resulted from the double impact of both recombination and mutation. However, as previously reported [12], and confirmed in the present study, evidence of recombination does indeed exist in STB HKG, albeit at very low rates compared to PE/PE_PGRS genes.

Our estimates indicate that both recombination and mutation contributed roughly equally to PE/PE_PGRS diversity. The intervention of both mechanisms is likely to be inherently associated with the nature of their gene sequences. Indeed, PE/PE_PGRS genes share extensive homologous regions which make them more prone to recombination than any other sequences [7]. We have previously reported on the occurrence of such rearrangements in MTBC strains either via gene conversion [22], involving neighboring PE_PGRS genes, or through intragenic and/or intergenic homologous recombination events [28]. In the present study we provide evidence for the occurrence of both types of recombination in STB. Strong intragenic recombination signals were observed in PE_PGRS51, a gene whose product was found to be the target of an antibody response [84]. Aside from PE_PGRS51, which is likely to represent a recombination hotspot, intragenic recombination could not be consistently demonstrated in the other PE/PE_PGRS genes. However, the fact that recombination was significantly detected when their sequences were concatenated, suggests their involvement in intergenic recombination events. In our previous study using a microarray targeting PE/PE_PGRS/PPE genes, 4 out of the five characterized recombination events were intergenic and involved neighboring PE/PE_PGRS/PPE genes [28]. Taken together, these findings indicate that intergenic recombination in PE/PE_PGRS sequences is likely to occur more frequently than intragenic recombination.

The relative high recombination signals observed in STB PE/PE_PGRS genes may not only reflect typical homologous recombination and gene conversion events, but may also include lateral transfer events. Indeed, STB have previously been shown to be the subject of episodes of horizontal gene transfer, some of which involved PE/PE_PGRS-containing loci [85]. Recent evidence strongly supports an environmental reservoir for STB [86], a characteristic that might increase their ability to acquire new genetic material through horizontal gene transfer. Such a “permissive” environment may also result in frequent intergenomic recombination between STB genotypes [12], [17], a mechanism thought to occur very rarely in the mammalian-adapted MTBC group. However, the recent finding in *M. tuberculosis* genomes of recombinant tracts matching *M. canettii* sequences lends further support that intergenomic recombination in *M. tuberculosis* might occur more frequently than previously thought [87].

Aside from homologous sequences, PE/PE_PGRS genes harbor a large number of sequence repeats, thus providing an optimal environment for rearrangements and mutations, which are direct consequences of the replication slippage phenomenon [88], [89]. Hence, aside from creating diversity, frequent recombination may also contribute to maintain sequence integrity against deleterious slip-strand mutations. Such a mechanism may be of importance for PE/PE_PGRS duplicates in particular. In a previous study we showed that PE_PGRS17 has acquired, most likely through horizontal gene transfer in the ancestor clone, a new DNA stretch which is then transferred to its neighboring paralogue, PE_PGRS18, through gene conversion [22]. We demonstrated that the reverse mechanism could take place naturally resulting in the elimination of the acquired DNA stretch from the PE_PGRS17 copy, thus restoring its original sequence. This previous study provided the proof of principle that recombination not only serves to generate diversity in PE_PGRS genes, but also contributes to prevent excessive sequence divergence that may lead to non-functionalization.

By virtue of their involvement in many facets of host-pathogen interaction, proliferation of PE/PE_PGRS genes and their expansion in the pathogenic MTBC could have provided the raw materials for functional innovations, essentially during the critical step of host adaptation. If the STB population represents a critical step in the evolution of mycobacterial pathogenicity, therefore imprints of adaptive evolution should be particularly evident in their PE/PE_PGRS gene sequences. Our findings confirm such hypothesis, since signals of positive selection operating on specific amino acid residues, or along branches of PE_PGRS phylogenetic trees, was demonstrated. In fact, aside from PE_PGRS26 and PE_PGRS62 where positive selection, with consistent dN and dS values, was evident for some branches (leading to genotypes A and I, respectively) (Fig. 5), the zero value of dS obtained with other branches may not allow to firmly argue for a true positive selection process. However, one should take into account, that with the covarion-based approach that we adopted for the branch model, dN values are calculated by sampling only those sites that are potentially under selective pressure. Therefore, irrespective of the dS value, even low dN values could be indicative of positive selection. The case of PE_PGRS62 deserves particular attention. While it proved polymorphic and positively selected in STB (in both “site” and “branch” models), this gene seems to evolve under a strict purifying selection in MTBC. Indeed, sequencing of 25 geographically diverse MTBC strains of different genotypes revealed its highly conserved sequence. In agreement with our findings, PE_PGRS62 was found remarkably conserved in a recent study describing the sequence variability of PE/PE_PGRS/PPE genes in present-day MTBC clinical isolates [31]. Strikingly, the ortholog of PE_PGRS62 in *M. marinum* has been shown to be critical for persistence in macrophages and in granuloma [40], a role that was further confirmed in BCG [44]. In human TB, patterns of seroreactivity to PE_PGRS62 were shown to correlate with clinical status and are associated with latent TB infection [90]. Recently, it was shown that expression of PE_PGRS62 in *M. smegmatis* resulted in reduced phagolysosome maturation in human macrophages, thus better discerning its role in persistence [91]. Hence, one is tempting to speculate that the positive selective pressure under which PE_PGRS62 evolved in STB could have contributed to endow it with critical functions, whose optimal coding sequence is likely to be fixed in present-day MTBC strains as a result of functional constraint. In this respect, it is worthy of mentioning that two of the amino acid changes that were positively selected (N253D and Q307H) in STB PE_PGRS62 could impact the protein conformation in their vicinity, since they involved the replacement of neutral polar amino acids with acidic and basic residues, respectively. No structural model of any PE_PGRS protein is available hitherto; therefore one can hardly predict the structural consequences of the positively selected amino acid changes.

A hallmark of TB epidemiology caused by STB consists in its high geographical clustering; all cases, but one, originated from the Horn of Africa or East African countries [16], [17]. This has led to the legitimate hypothesis that STB, although cause pulmonary TB, could not be transmitted between humans. It has thus been speculated that STB-associated pulmonary TB could be a consequence of aerosol exposure from environmental sources, and evidence supporting such a hypothesis have been recently presented [86]. Should this be the case, clustered TB cases due to STB are likely to represent patients that have been exposed to the same environmental source. In the present study, we found that STB strains belonging to the same genotypes have identical PE/PE_PGRS nucleotide sequences. This finding confirms the data obtained by Gutierrez et al. [12], according to which there were no sequence variations in HKG genes between strains of the same genotype. If STB indeed prove impaired in their ability to be transmitted between humans, then the highest prevalence of certain genotypes [16], like genotype C/D, may indicate that they are more prone than others to cause disease in humans. Therefore, if transmission does indeed occur through an environmental host, the signals of positive selection detected in PE/PE_PGRS genes are likely to reflect a combination of sites that confer increased ability to cause overt TB in humans. Consequently, one would expect for much higher diversity in the STB population that is directly isolated from the environment.

## Conclusions

The data presented in this study point to a high rate of genetic remodeling in STB PE/PE_PGRS genes, owing to the double contribution of recombination and mutation, with evidence of positive selection. Recombination is likely to have accelerated the diversification process of STB PE/PE_PGRS genes, by introducing an excess of nonsynonymous mutations, thus providing the raw material allowing selection to operate. This study also provides an obvious example of a PE_PGRS gene, PE_PGRS62, which is subject to positive selection in STB and which could have been driven to fixation in present-day MTBC strains, as reflected by its highly conserved nature. Such a finding is consistent with previous reports highlighting the critical role of this PE_PGRS gene in both the replication and persistence of the bacillus. Overall this study stresses the need to further explore the evolution of PE/PE_PGRS genes in the ancestor of the MTBC, as they may hold the key to understanding the transition of mycobacteria from the environment to mammalian hosts.

## Supporting Information

Table S1 Strains of smooth tubercle bacilli used in this Study.
(DOCX)

Table S2 Oligonucleotide primers used for PCR amplification and sequencing of PE/PE_PGRS gene fragments.
(DOCX)

Table S3 Characteristics of *M. tuberculosis* strains used to extend the polymorphism analysis of PE_PGRS62.
(DOCX)

Table S4 Compilation of the results obtained with the various recombination detection tests
(DOCX)

File S1 **The best substitution models obtained with TOPAli.**
(PDF)
