# Supplementary material for: Evolution of Smooth Tubercle Bacilli PE and PE_PGRS Genes: Evidence for a Prominent Role of Recombination and Imprint of Positive Selection
Source: PLoS One. 2013 May 21;8(5):e64718. doi: 10.1371/journal.pone.0064718 (PMC3660525; doi:10.1371/journal.pone.0064718)
Supplement: Table S1 — Strains of smooth tubercle bacilli used in this Study. (DOCX) [file pone.0064718.s001.docx]

**Table S1.** Strains of smooth tubercle bacilli used in this Study.

| **Strain number** | **Strain code** | **TB site** | **Year of isolation** | **Country of isolation** | **Group*** |
| --- | --- | --- | --- | --- | --- |
| 1 | 140010060 | Pulmonary | 1969 | France | A |
| 2 | 19990160 | Pulmonary | 1999 | France | C/D |
| 3 | 19990161 | Lymph node | 1999 | Djibouti | C/D |
| 8 | 19990516 | Pulmonary | 1999 | Djibouti | C/D |
| 9 | 19990515 | Pulmonary | 1999 | Djibouti | C/D |
| 10 | 19990589 | Pulmonary | 1999 | Djibouti | C/D |
| 11 | 19990711 | Lymph node | 1999 | Djibouti | B |
| 14 | 20010933 | Lymph node | 2001 | Djibouti | C/D |
| 15 | 20001049 | Pulmonary | 2000 | Djibouti | C/D |
| 16 | 20001245 | Pulmonary | 2000 | Djibouti | C/D |
| 18 | 20001247 | Peritoneal liquid | 2000 | Djibouti | C/D |
| 20 | 19981514 | Lymph node | 1998 | Djibouti | C/D |
| 22 | 19991704 | Pulmonary | 1999 | Djibouti | C/D |
| 25 | 19991709 | Lymph node | 1999 | Djibouti | E |
| 26 | 20000342 | ND | 2000 | Djibouti | C/D |
| 27 | 20000587 | Lymph node | 2000 | Djibouti | G |
| 29 | 19980863 | Pulmonary | 1998 | Djibouti | H |
| 30 | 19980864 | Pulmonary | 1998 | Djibouti | H |
| 31 | 20020544 | Pulmonary | 2002 | Djibouti | C/D |
| 32 | 20000586 | Pulmonary | 2000 | Djibouti | C/D |
| 34 | 20020986 | Lymph node | 2002 | Djibouti | C/D |
| 37 | 20020989 | Pulmonary | 2002 | Djibouti | C/D |
| 41 | 20030467 | Blood | 2003 | Djibouti | C/D |
| 44 | 20030686 | Blood | 2003 | Djibouti | C/D |
| 45 | 20031437 | Lymph node | 2003 | Djibouti | C/D |
| 47 | 20041158 | Skin | 2004 | Djibouti | I |
| 48 | 20050462 | Lymph node | 1997 | Djibouti | F |
| 49 | 20050642 | Pulmonary | 2005 | France | C/D |
|  |  |  |  |  |  |

* As determined by MIRU-VNTR 24 [11,12].
