# Supplementary material for: Evolution of Smooth Tubercle Bacilli PE and PE_PGRS Genes: Evidence for a Prominent Role of Recombination and Imprint of Positive Selection
Source: PLoS One. 2013 May 21;8(5):e64718. doi: 10.1371/journal.pone.0064718 (PMC3660525; doi:10.1371/journal.pone.0064718)
Supplement: Table S2 — Oligonucleotide primers used for PCR amplification and sequencing of PE/PE_PGRS gene fragments. (DOCX) [file pone.0064718.s002.docx]

**Table S2.** Oligonucleotide primers used for PCR amplification and sequencing of PE/PE_PGRS gene fragments.

| **Gene** | **Name** | **Sequence (5’-3’)** |
| --- | --- | --- |
| PE3 (Rv0159c) | 0159S1  0159S2* | TGGCAACGACGGCCGCGGAC  GTTTTCGGCTACTCGCAAAG |
|  | 0159R1  0159R2* | AATCAATCTCGCTACGGACC  TTGGCCGGTGTACATGTAGG |
| PE4 (Rv0160c) | 0160S1 | TGGTCACAGCCCCTGACATG |
|  | 0160S2*  0160S3* | TGTTCACGCCCGAGCAGTTC  GGAACTCATGGCCACCCAGG |
|  | 0160R1  0160R2* | TGCAGGATGTTGCCGAGCCA  ACTACCAATCATGACGAAGG |
| PE_PGRS12 (Rv0832) | 0832S1 | TGAGCGTGTTGCCCGCTACG |
|  | 0832R1 | CCGGTGCCCGGCTGGCCGTT |
| PE_PGRS26(Rv1441c) | 1441S1  1441S2* | ATGTCGAACGTGATGGTAGT  GACAGTCCTAACAGCGGTGC |
|  | 1441R1  1441R2* | TCACCCGTGCTTTCCTTGCG  CCGAACATCCCAGCAGCACC |
| PE_PGRS29(Rv1468c) | 1468S1  1468S2* | ATGTCGTTCGTGGTCGCGAA  GCAACGGCTTCTCGCCCGCT |
|  | 1468R1  1468R2* | TGTTCCGTTCGCGCCGGGCA  GCCGTTGCCGAACAGCCACC |
| PE_PGRS35(Rv1983) | 1983S1 | GTGTCATTTCTGGTCGTGGT |
|  | 1983S2*  1983R1  1983R2*  1983R3* | CCTGTTGTATGGCGGATACG  TCACGCCGGATGATCAAAGA  ACGATGCCATTCCCGAAGTC  TCCGCCGGCTCCACCACTCAT |
| PE_PGRS51(Rv3367) | 3367S1 | ATGTCGTTTGTCGTAGCAGT |
|  | 3367S2* | TGGTTGTACGGCAACGGCGG |
|  | 3367S3*  3367R1  3367R2*  3367R3* | GGTTGGTTGATGGGCAACGG  TTAGCCGGGGTTGCCGTCGG  GCTGTTACCGCCCTGGTTGG  CCGCCGTTGCCGATCAATCC |
| PE_PGRS62(Rv3812) | 3812S1  3812S2*  3812S3* | GTGTCGTTCGTGGTCACAGT  CTGGTGGCGAATTTGCCAGC  TCGTTCGATATCGCAACCAC |
|  | 3812R1  3812R2* | CTAAGCCGCCGGTTTGATTGC  ACCGCGGCGTTGTAGTCACC |
| PE35(Rv3872) | 3872S1 | ACAGGGAGGGAAGAAGTAGG |
|  | 3872R1 | CACTCCCTCCGATGTGTTGG |
|  |  |  |

*Internal primers used for sequencing
