# Supplementary material for: Evolution of Smooth Tubercle Bacilli PE and PE_PGRS Genes: Evidence for a Prominent Role of Recombination and Imprint of Positive Selection
Source: PLoS One. 2013 May 21;8(5):e64718. doi: 10.1371/journal.pone.0064718 (PMC3660525; doi:10.1371/journal.pone.0064718)
Supplement: Table S3 — Characteristics of M. tuberculosis strains used to extend the polymorphism analysis of PE_PGRS62. (DOCX) [file pone.0064718.s003.docx]

**Table S3.** Characteristics of *M. tuberculosis* strains used to extend the polymorphism analysis of PE_PGRS62.

| Designation | Origin | PGG^a^ | TbD1^b^ | Spoligotype family^c^ |
| --- | --- | --- | --- | --- |
| M.tb 1362/04 | TUNISIA | 2 | - | T1 |
| M.tb 1612/02 | TUNISIA | 2 | - | T2-T4 |
| M.tb 381/02 | TUNISIA | 2 | - | Haarlem3 |
| M.tb 662/03 | TUNISIA | 2 | - | orphan |
| M.tb 1181/02 | TUNISIA | 2 | - | LAM9 |
| M.tb 1695/01 | TUNISIA | 2 | - | Haarlem3 |
| M.tb 1659 | SOUTH AFRICA | 1 | + | EAI5 |
| M.tb 5867 BE | UNITED STATES | 1 | + | EAI4-Vietnam |
| M.tb 1592 N2 | UNITED STATES | 1 | - | Beijing |
| M.tb 13180 BW33 | PUERTO RICO | 2 | - | Haarlem3 |
| M.tb 10367 C | UNITED STATES | 2 | - | X3 |
| M.tb 11797 AI 10 | RUSSIA | 2 | - | LAM9 |
| M.tb 11074 AF52 | UNITED STATES | 3 | - | T1 |
| M.tb 13494 CS46 | UNITED STATES | 2 | - | LAM2 |
| M.tb 12657 KQ 15 | RUSSIA | 2 | - | Haarlem3 |
| M.tb 1956 | SOUTH AFRICA | 3 | - | T1 |
| M.tb 14862 KF15 | UNITED STATES | 1 | + | EAI2-Manilla |
| M.tb 14868 MB5 | UNITED STATES | 1 | - | CAS1-Delhi |
| M.tb 13984 NU | UNITED STATES | 1 | - | CAS1-Delhi |
| M.tb 14222 DN3 | UNITED STATES | 1 | - | Beijing |
| M.tb 14565 HE8 | UNITED STATES | 1 | - | Beijing |
| M.tb 14255 KQ37 | UNITED STATES | 2 |  | LAM9 |
| M.tb 13957 MB4 | UNITED STATES | 1 | - | CAS1-Delhi |
| M.tb 9139 AF | UNITED STATES | 3 | - | T1 |
| M.tb 14214 001 | UNITED STATES | 1 | - | Beijing |

^a^PGG : principal genetic group according to *katG*463 and *gyrA*95 polymorphism as defined by Sreevatsan et al. [6].

^b^A genomic region according to which *M. tuberculosis* strains can be divided into « ancestral » (not deleted ; +) and « modern » strains (deleted ; -) [2].

^c^As defined by the polymorphism in the DR region. Spoligotype family was assigned according to SITVIT WEB (www.pasteur-guadeloupe.fr:8081/SITVIT_ONLINE/).
