# Supplementary material for: Evolution of Smooth Tubercle Bacilli PE and PE_PGRS Genes: Evidence for a Prominent Role of Recombination and Imprint of Positive Selection
Source: PLoS One. 2013 May 21;8(5):e64718. doi: 10.1371/journal.pone.0064718 (PMC3660525; doi:10.1371/journal.pone.0064718)
Supplement: Table S4 — Compilation of the results obtained with the various recombination detection tests (DOCX) [file pone.0064718.s004.docx]

**Table S4.** Compilation of the results obtained with the various recombination detection tests.

| **Recombination detection test** | **PE3** | **PE4** | **PE_PGRS26** | **PE_PGRS35** | **PE_PGRS51** | **Concatenated gene sequences** |
| --- | --- | --- | --- | --- | --- | --- |
|  |  |  |  |  |  |  |
| Hudson and Kaplan’s *R*_min_ | 0 | 2 | 1 | 1 | 2 | 10 |
| (breakpoints position) |  | (**533**,1016) | (**504**,1328) | (**660**,1248) | (304,809) | (465, 1820) |
|  |  | (1016,1052) |  |  | (**1198**,1290) | (1862, 2325) |
|  |  |  |  |  |  | (2325, 2361) |
|  |  |  |  |  |  | (2361, 2908) |
|  |  |  |  |  |  | (3240, 4064) |
|  |  |  |  |  |  | (4064, 4706) |
|  |  |  |  |  |  | (4799, 5387) |
|  |  |  |  |  |  | (5387, 5847) |
|  |  |  |  |  |  | (6062, 6567) |
|  |  |  |  |  |  | (6959, 7048) |
|  |  |  |  |  |  |  |
| Maximum chi-square | No | No | No | No | No | Yes |
|  |  |  |  |  |  |  |
| Recco (*P* value)^*^ | No (1.0) | No (0.43) | No (0.32) | No (0.79) | Yes (0.006) | No (0.25) |
|  |  |  |  |  |  |  |
| GARD | No | 1 | 1 | 1 | 2 | 5 |
| (breakpoints position) |  | (**533**, **1016**) | (**504**) | (**660**) | (623, **1198**) | (1620, 3706, 5387, 6381, 6956) |
|  |  |  |  |  |  |  |
| PHI test (*p*-value) | 1.0 | 0.18 | 0.2 | 0.11 | **0.011** | **3.17E-4** |
|  |  |  |  |  |  |  |

*The probability of detecting recombination feature of Recco was adjusted such as to classify a dataset as recombinant if the *P*-value does not exceed 0.12.

Recombinant segments or breakpoints identified in both single gene or concatenated sequence analyses are indicated with the same color. Identical breakpoints shared with both Hudson and Kaplan’s *R*_min_ as well as GARD are highlighted in bold. Breakpoints detected by GARD that were model supported are underlined. For PHI test, a *p*-value lower or equal 0.05 reflect presence of recombination.

Note that recombination signals associated with PE_PGRS51 was detected by 4 of the 5 algorithms.
