# Supplementary material for: Evolution of Smooth Tubercle Bacilli PE and PE_PGRS Genes: Evidence for a Prominent Role of Recombination and Imprint of Positive Selection
Source: PLoS One. 2013 May 21;8(5):e64718. doi: 10.1371/journal.pone.0064718 (PMC3660525; doi:10.1371/journal.pone.0064718)

PE3

Model	K	$-\ell$	AIC_1	AIC_2	BIC ▲
**F81**	3	1891,15	3814,30 (0)	3814,72 (0)	3897,19 (0)
F81+I	4	1890,42	3814,84 (0)	3815,32 (0)	3902,92 (0)
HKY	4	1891,11	3816,21 (0)	3816,68 (0)	3904,29 (0)
TrN	5	1889,80	3815,59 (0)	3816,12 (0)	3908,85 (0)
K81uf	5	1889,92	3815,84 (0)	3816,36 (0)	3909,09 (0)
HKY+I	5	1890,38	3816,76 (0)	3817,28 (0)	3910,01 (0)
TIM	6	1888,61	3815,22 (0)	3815,80 (0)	3913,65 (0)
TrN+I	6	1889,07	3816,14 (0)	3816,72 (0)	3914,57 (0)
K81uf+I	6	1889,19	3816,38 (0)	3816,97 (0)	3914,82 (0)
TIM+I	7	1887,88	3815,76 (0)	3816,41 (0)	3919,38 (0)
TVM	7	1888,73	3817,45 (0)	3818,10 (0)	3921,07 (0)
GTR	8	1887,49	3816,97 (0)	3817,69 (0)	3925,77 (0)
TVM+I	8	1888,00	3818,01 (0)	3818,72 (0)	3926,81 (0)
GTR+I	9	1886,76	3817,52 (0)	3818,30 (0)	3931,50 (0)
JC	0	1937,09	3900,19 (0)	3900,47 (0)	3967,54 (0)
JC+I	1	1936,37	3900,74 (0)	3901,06 (0)	3973,27 (0)
K80	1	1937,08	3902,15 (0)	3902,48 (0)	3974,68 (0)
K81	2	1935,61	3901,21 (0)	3901,58 (0)	3978,92 (0)
TrNef	2	1935,91	3901,82 (0)	3902,18 (0)	3979,53 (0)
K80+I	2	1936,35	3902,71 (0)	3903,08 (0)	3980,42 (0)
TIMef	3	1934,44	3900,87 (0)	3901,29 (0)	3983,77 (0)

Select as default model for this alignment

F81 (Felsenstein,1981)

Rate Heterogeneity (Γ): α = n/a

Proportion of Invariant Sites: pINV = n/a

Substitution Rates: 1,000 | 1,000 | 1,000 | 1,000 | 1,000 | 1,000 (000000)

Base Frequencies: 0,186 | 0,343 | 0,282 | 0,188 (0123)

Scores:

ℓ = −1891,15; AIC_1 = 3814,30; AIC_2 = 3814,72; BIC = 3897,19

(df = 16; n = 1314)

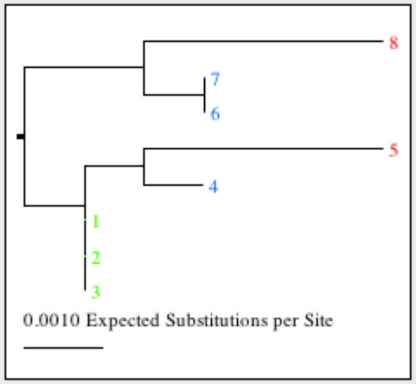

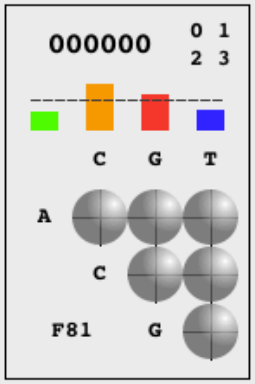

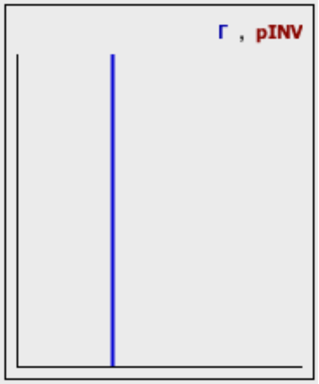

PE4

Model	K	$-\ell$	AIC_1	AIC_2	BIC
**HKY**	4	2085,46	4204,93 (0)	4205,36 (0)	4294,45 (0)
TrN	5	2082,82	4201,64 (0)	4202,13 (0)	4296,43 (0)
F81	3	2090,11	4212,22 (0)	4212,60 (0)	4296,48 (0)
HKY+I	5	2084,35	4204,69 (0)	4205,18 (0)	4299,48 (0)
TrN+I	6	2081,71	4201,42 (0)	4201,96 (0)	4301,47 (0)
F81+I	4	2089,00	4211,99 (0)	4212,43 (0)	4301,52 (0)
K81uf	5	2086,42	4208,84 (0)	4209,32 (0)	4303,63 (0)
TIM	6	2082,80	4203,60 (0)	4204,14 (0)	4303,66 (0)
K81uf+I	6	2085,30	4208,60 (0)	4209,14 (0)	4308,66 (0)
TIM+I	7	2081,69	4203,38 (0)	4203,98 (0)	4308,70 (0)
TVM	7	2083,39	4206,78 (0)	4207,38 (0)	4312,10 (0)
GTR	8	2081,11	4204,22 (0)	4204,88 (0)	4314,81 (0)
TVM+I	8	2082,25	4206,51 (0)	4207,16 (0)	4317,10 (0)
GTR+I	9	2080,08	4204,15 (0)	4204,87 (0)	4320,01 (0)
K80	1	2140,63	4309,27 (0)	4309,56 (0)	4382,99 (0)
JC	0	2144,81	4315,61 (0)	4315,87 (0)	4384,07 (0)
TrNef	2	2138,24	4306,48 (0)	4306,82 (0)	4385,48 (0)
K80+I	2	2139,52	4309,05 (0)	4309,39 (0)	4388,04 (0)
JC+I	1	2143,70	4315,39 (0)	4315,69 (0)	4389,12 (0)
K81	2	2140,56	4311,12 (0)	4311,46 (0)	4390,11 (0)
TrNef+I	3	2137,20	4306,40 (0)	4306,78 (0)	4390,65 (0)

Select as default model for this alignment

HKY (HKY85; Hasegawa, Kishino and Yano,1985)

Rate Heterogeneity (Γ): α = n/a
Proportion of Invariant Sites: pINV = n/a
Substitution Rates: 1,000 | 4,006 | 1,000 | 1,000 | 4,006 | 1,000 (010010)
Base Frequencies: 0,182 | 0,344 | 0,291 | 0,183 (0123)
Scores:
$\ell = -2085{,}46$; $AIC_1 = 4204{,}93$; $AIC_2 = 4205{,}36$; BIC = 4294,45
(df = 17; n = 1431)

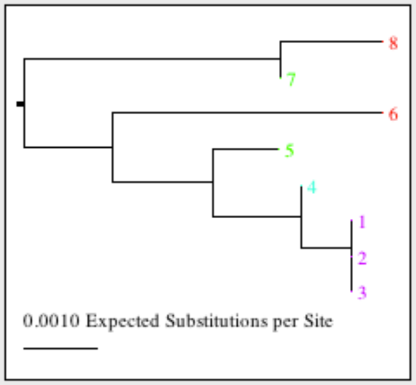

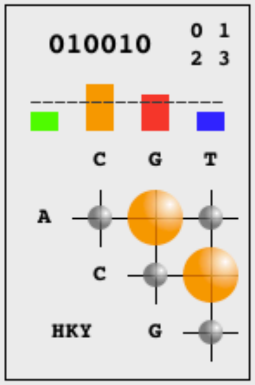

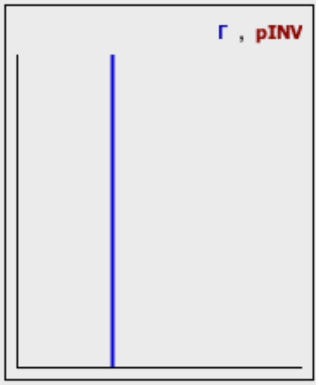

PE_PGRS26

Model	K	$-\ell$	AIC_1	AIC_2	BIC
**HKY**	4	1889,06	3812,12 (0)	3812,56 (0)	3901,43 (0)
TrN	5	1885,62	3807,25 (0)	3807,74 (0)	3901,81 (0)
HKY+I	5	1888,84	3813,67 (0)	3814,16 (0)	3908,23 (0)
TrN+I	6	1885,41	3808,82 (0)	3809,36 (0)	3908,63 (0)
K81uf	5	1889,06	3814,11 (0)	3814,60 (0)	3908,67 (0)
TIM	6	1885,62	3809,24 (0)	3809,79 (0)	3909,06 (0)
K81uf+I	6	1888,83	3815,67 (0)	3816,21 (0)	3915,48 (0)
TIM+I	7	1885,41	3810,81 (0)	3811,42 (0)	3915,88 (0)
TVM	7	1887,16	3814,32 (0)	3814,92 (0)	3919,39 (0)
GTR	8	1885,02	3812,03 (0)	3812,70 (0)	3922,36 (0)
TVM+I	8	1886,94	3815,87 (0)	3816,54 (0)	3926,19 (0)
F81	3	1906,43	3844,86 (0)	3845,25 (0)	3928,91 (0)
GTR+I	9	1884,81	3813,63 (0)	3814,35 (0)	3929,20 (0)
F81+I	4	1906,21	3846,42 (0)	3846,86 (0)	3935,73 (0)
K80	1	2119,87	4267,75 (2)	4268,05 (2)	4341,30 (2)
TrNef	2	2117,59	4265,18 (2)	4265,52 (2)	4343,98 (2)
K80+I	2	2119,64	4269,27 (2)	4269,62 (2)	4348,08 (2)
K81	2	2119,70	4269,40 (2)	4269,75 (2)	4348,21 (2)
TrNef+I	3	2117,40	4266,79 (2)	4267,18 (2)	4350,85 (2)
TIMef	3	2117,42	4266,83 (2)	4267,22 (2)	4350,89 (2)
K81+I	3	2119,46	4270,93 (2)	4271,32 (2)	4354,98 (2)

Select as default model for this alignment

HKY (HKY85; Hasegawa, Kishino and Yano,1985)

Rate Heterogeneity (Γ): α = n/a
Proportion of Invariant Sites: pINV = n/a
Substitution Rates: 1,000 | 17,560 | 1,000 | 1,000 | 17,560 | 1,000 (010010)
Base Frequencies: 0,127 | 0,340 | 0,431 | 0,102 (0123)
Scores:
$\ell = -1889{,}06$; $AIC_1 = 3812{,}12$; $AIC_2 = 3812{,}56$; BIC = 3901,43
(df = 17; n = 1413)

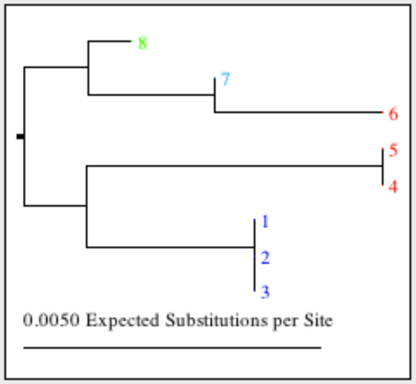

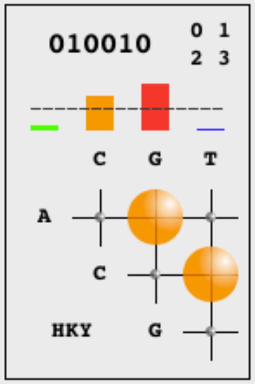

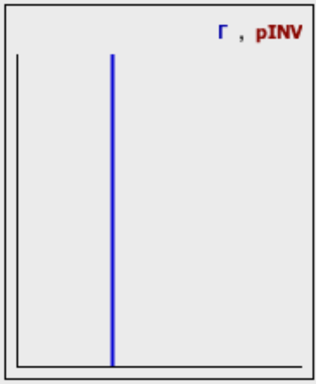

PE_PGRS35

Model	K	−ℓ	AIC_1	AIC_2	BIC
**F81**	3	2272,08	4576,17 (0)	4576,51 (0)	4662,41 (0)
F81+I	4	2271,64	4577,28 (2)	4577,66 (2)	4668,91 (2)
HKY	4	2272,08	4578,17 (0)	4578,55 (0)	4669,80 (0)
HKY+I	5	2271,64	4579,28 (2)	4579,71 (2)	4676,30 (2)
TrN	5	2272,08	4580,17 (0)	4580,59 (0)	4677,19 (0)
K81uf	5	2272,08	4580,17 (0)	4580,59 (0)	4677,19 (0)
TrN+I	6	2271,64	4581,28 (2)	4581,76 (2)	4683,69 (2)
K81uf+I	6	2271,64	4581,28 (2)	4581,76 (2)	4683,69 (2)
TIM	6	2272,08	4582,17 (0)	4582,64 (0)	4684,58 (0)
TIM+I	7	2271,64	4583,28 (2)	4583,81 (2)	4691,08 (2)
TVM	7	2272,08	4584,17 (0)	4584,69 (0)	4691,97 (0)
TVM+I	8	2271,64	4585,28 (2)	4585,86 (2)	4698,47 (2)
GTR	8	2272,08	4586,17 (0)	4586,75 (0)	4699,36 (0)
GTR+I	9	2271,64	4587,28 (2)	4587,91 (2)	4705,86 (2)
JC	0	2350,81	4727,61 (2)	4727,84 (2)	4797,68 (2)
JC+I	1	2350,36	4728,73 (0)	4728,99 (0)	4804,19 (0)
K80	1	2350,81	4729,61 (2)	4729,87 (2)	4805,07 (2)
K80+I	2	2350,36	4730,73 (0)	4731,02 (0)	4811,58 (0)
TrNef	2	2350,81	4731,61 (2)	4731,91 (2)	4812,46 (2)
K81	2	2350,81	4731,61 (2)	4731,91 (2)	4812,46 (2)
TrNef+I	3	2350,36	4732,73 (0)	4733,06 (0)	4818,97 (0)

Select as default model for this alignment

F81 (Felsenstein,1981)

Rate Heterogeneity (Γ): α = n/a
Proportion of Invariant Sites: pINV = n/a
Substitution Rates: 1,000 | 1,000 | 1,000 | 1,000 | 1,000 | 1,000 (000000)
Base Frequencies: 0,170 | 0,313 | 0,341 | 0,175 (0123)
Scores:
ℓ = −2272,08; AIC_1 = 4576,17; AIC_2 = 4576,51; BIC = 4662,41
(df = 16; n = 1620)

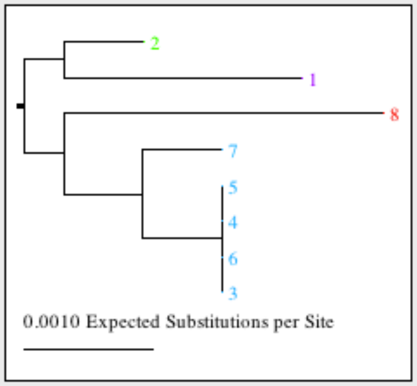

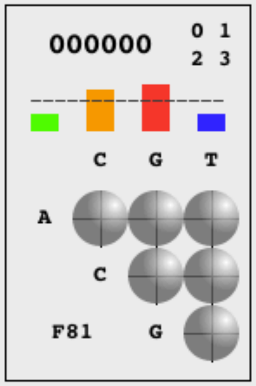

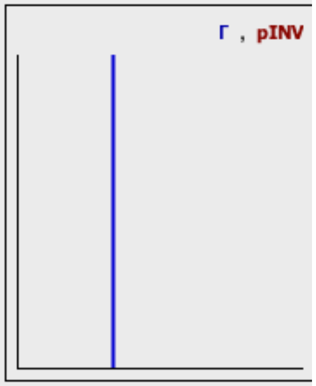

PE_PGRS51

Model	K	$-\ell$	AIC_1	AIC_2	BIC
TrN+I	6	2488,27	5014,54 (0)	5014,99 (0)	5117,94 (0)
K81uf+I	6	2488,67	5015,34 (0)	5015,79 (0)	5118,75 (0)
TrN	5	2492,53	5021,05 (0)	5021,46 (0)	5119,02 (0)
K81uf	5	2492,92	5021,85 (0)	5022,26 (0)	5119,81 (0)
TVM+I	8	2483,63	5009,26 (0)	5009,80 (0)	5123,55 (0)
TIM+I	7	2487,74	5015,49 (0)	5015,99 (0)	5124,34 (0)
TVM	7	2487,90	5015,81 (0)	5016,31 (0)	5124,66 (0)
TIM	6	2492,01	5022,03 (0)	5022,48 (0)	5125,44 (0)
HKY+I	5	2496,80	5029,60 (0)	5030,01 (0)	5127,57 (0)
HKY	4	2501,13	5036,26 (0)	5036,62 (0)	5128,78 (0)
GTR+I	9	2485,15	5014,29 (0)	5014,89 (0)	5134,03 (0)
GTR	8	2489,35	5020,71 (0)	5021,26 (0)	5135,00 (0)
F81+I	4	2506,35	5046,70 (0)	5047,06 (0)	5139,22 (0)
F81	3	2510,54	5053,09 (0)	5053,41 (0)	5140,17 (0)
TrNef+I	3	2724,22	5480,44 (0)	5480,77 (0)	5567,52 (0)
TrNef	2	2728,34	5486,69 (0)	5486,97 (0)	5568,33 (0)
TVMef+I	5	2717,89	5471,78 (0)	5472,19 (0)	5569,75 (0)
TVMef	4	2722,33	5478,65 (0)	5479,01 (0)	5571,17 (0)
K81+I	3	2727,85	5487,70 (0)	5488,02 (0)	5574,78 (0)
TIMef+I	4	2724,22	5482,44 (0)	5482,81 (0)	5574,97 (0)

Select as default model for this alignment

TrN (TN; TN93; Tamura and Nei, 1993)

Rate Heterogeneity (Γ): α = n/a

Proportion of Invariant Sites: pINV = 0,349

Substitution Rates: 1,000 | 4,510 | 1,000 | 1,000 | 7,235 | 1,000 (010020)

Base Frequencies: 0,149 | 0,330 | 0,416 | 0,104 (0123)

Scores:

ℓ = −2488,27; AIC_1 = 5014,54; AIC_2 = 5014,99; BIC = 5117,94

(df = 19; n = 1707)

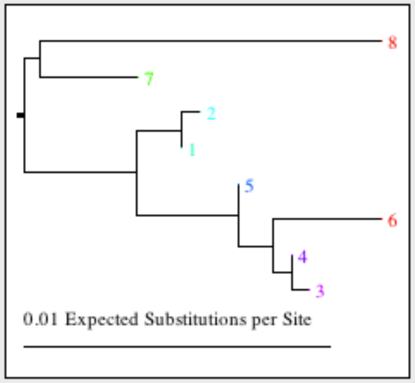

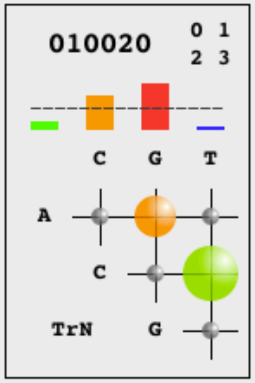

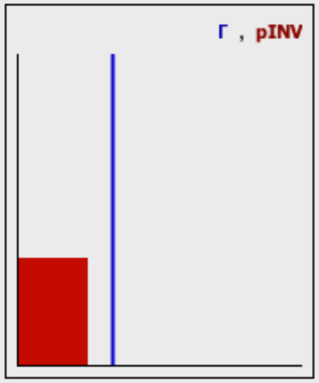

PE_PGRS62

Model	K	$-\ell$	AIC_1	AIC_2	BIC
**F81**	3	1977,49	3986,98 (0)	3987,36 (0)	4071,40 (0)
HKY	4	1977,49	3988,98 (0)	3989,41 (0)	4078,67 (0)
F81+I	4	1977,49	3988,98 (0)	3989,41 (0)	4078,67 (0)
TrN	5	1977,49	3990,98 (0)	3991,46 (0)	4085,95 (0)
K81uf	5	1977,49	3990,98 (0)	3991,46 (0)	4085,95 (0)
HKY+I	5	1977,49	3990,98 (0)	3991,46 (0)	4085,95 (0)
TIM	6	1977,49	3992,98 (0)	3993,51 (0)	4093,22 (0)
TrN+I	6	1977,49	3992,98 (0)	3993,52 (0)	4093,23 (0)
K81uf+I	6	1977,49	3992,98 (0)	3993,52 (0)	4093,23 (0)
TVM	7	1977,49	3994,98 (0)	3995,57 (0)	4100,50 (0)
TIM+I	7	1977,49	3994,98 (0)	3995,57 (0)	4100,50 (0)
GTR	8	1977,49	3996,98 (0)	3997,63 (0)	4107,77 (0)
TVM+I	8	1977,49	3996,98 (0)	3997,63 (0)	4107,78 (0)
GTR+I	9	1977,49	3998,98 (0)	3999,70 (0)	4115,05 (0)
JC	0	2039,30	4104,61 (0)	4104,86 (0)	4173,20 (0)
K80	1	2039,30	4106,61 (0)	4106,90 (0)	4180,47 (0)
JC+I	1	2039,31	4106,61 (0)	4106,91 (0)	4180,47 (0)
TrNef	2	2039,30	4108,61 (0)	4108,95 (0)	4187,75 (0)
K81	2	2039,30	4108,61 (0)	4108,95 (0)	4187,75 (0)
K80+I	2	2039,31	4108,61 (0)	4108,95 (0)	4187,75 (0)

Select as default model for this alignment

F81 (Felsenstein,1981)

Rate Heterogeneity (Γ): α = n/a

Proportion of Invariant Sites: pINV = n/a

Substitution Rates: 1,000 | 1,000 | 1,000 | 1,000 | 1,000 | 1,000 (000000)

Base Frequencies: 0,177 | 0,351 | 0,288 | 0,183 (0123)

Scores:

ℓ = −1977,49; AIC_1 = 3986,98; AIC_2 = 3987,36; BIC = 4071,40 (df = 16; n = 1445)

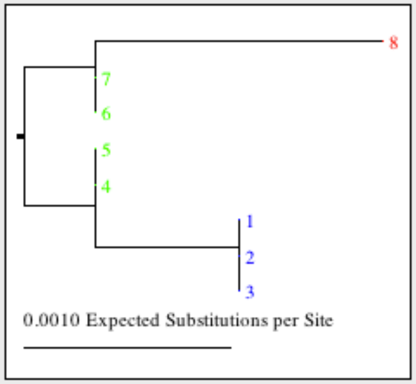

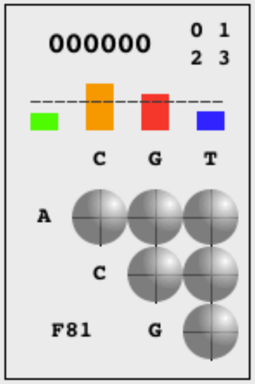

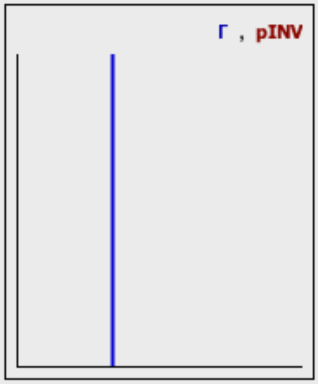

Supplement: File S1 — The best substitution models obtained with TOPAli. (PDF) [file pone.0064718.s005.pdf]
